# HVSnp: A manually curated database of human SNPS associated with viral infections

**DOI:** 10.1590/1678-4685-GMB-2025-0075

**Published:** 2026-10-19

**Authors:** Ana Carolina Laureano Paganin, Caio César de Melo Freire

**Affiliations:** 1 Universidade Federal de São Carlos, Departamento de Genética e Evolução, São Carlos, SP, Brazil

**Keywords:** Database, polymorphisms, virus, viral disease

## Abstract

We developed the “HVSnp: Human Viruses SNP-related database, a curated database”.* *HVSnp is exclusive to the human SNPs studied and related to viral diseases, with manually mined and curated information from public SNP databases and scientific articles. We used MySQL (version 8.0) due to the easy management and the Looker Studio online availability. Among the output information, we have SNP identification, reference, alternative, and risk alleles, chromosome, gene, and gene function, summary, among others. In total, HVSnp contains eighteen viral diseases and 2020 records: Adenovirus (17 SNPs), Chikungunya (7), COVID-19 (314), Cytomegalovirus (54), Dengue (62), Epstein-Barr (54), Hepatitis A (3), Hepatitis B (380), Hepatitis C (166), Herpes (8), Human immunodeficiency virus (479), Papillomavirus (87), Influenza (74), Measles (68), Respiratory syncytial virus (50), Sars-CoV (80), West Nile (68), and Zika (49). As one SNP can be associated with more than one virus, the number of unique SNPs is 1691 and 651 genes. HVSnp has supplementary information obtained in articles for the summary elaboration, which contains the most significant results described in the literature about a possible relationship between SNPs and viruses. The database is available at URL: https://lookerstudio.google.com/s/pGUC7m538gQ.

## Introduction

Viruses are the largest and most genetically diversified biological entities on Earth (Cobián Güemes et al., 2016; Paez-Espino et al., 2016). However, approximately 270 viruses are recognized to infect humans (Forni et al., 2022). Some human viruses have a known zoonotic origin, such as Human immunodeficiency virus (HIV), Ebola virus (EBOV), Chikungunya virus (CHIKV), Dengue (DENV), Zika virus (ZIKV), Influenza A (IA), Severe acute respiratory syndrome (SARS), and Severe acute respiratory syndrome coronavirus 2 (SARS-CoV-2) (Mandl et al., 2015; Forni *et al.,* 2022). Emerging and re-emerging infectious disease outbreaks were frequent in the 21^th^ century. For example, during the 2003 SARS outbreak, the 2009 H1N1 influenza (swine flu) pandemic, the 2012 Middle East respiratory syndrome coronavirus (MERS-CoV) outbreak, from 2013 to 2016 EBOV disease epidemic in West Africa, and 2018 in the Democratic Republic of Congo, the 2015 ZIKV epidemic, and the 2019 Coronavirus disease 2019 (COVID-19) caused by SARS-CoV-2. (Ellwanger et al., 2021; Wilder-Smith 2021; Baker et al., 2022).

Much of the efforts to investigate viral pathogenesis are based on virulence elements. However, virulence can only be defined in the context of viral-host interaction (Ferris et al., 2016); genetic studies are essential to identify biological mechanisms related to the susceptibility to several diseases or conditions, discover targets of therapeutic purpose, and even explain causal relationships that can modify some environmental risk aspects (Cappadona et al., 2023). Several genes directly impact the adaptive immune response and biological activities such as transcription regulation and intracellular transport. The chemokine receptor 2 (CCR2) gene, for example, is responsible for recruiting basophils, monocytes, and T cells to sites of inflammatory processes. It played a defensive role against CHIKV and helped improve pathology in influenza virus (Poo et al., 2014; Ferris *et al.,* 2016).

The global prevalence of HIV infection, which causes acquired immunodeficiency syndrome (AIDS), is high, even with a decrease in new cases compared to the beginning of this century (Maartens et al., 2014). Due to its socioeconomic impact, studies associated with viral genetic diversity, which can contribute to improving the resistance and infectivity of the virus, have been carried out, and the mining of human single nucleotide polymorphisms (SNPs) related to HIV infection and disease progression can support the development of new therapies and vaccines (McLaren and Carrington, 2015; Tough and McLaren, 2019).

Genome-wide association studies (GWAS) have identified polymorphisms related to viral infection susceptibility in large patient cohorts. Other techniques to study polymorphisms are whole exome sequencing (WES) and candidate genes (Kenney et al., 2017). SNPs are the most frequent type of variation in the genomic sequence. They are recognized as valuable genetic markers for indicating evolutionary aspects and common genetic polymorphisms, which contribute to understanding the heritable risk for diseases (Fareed and Afzal 2013). The growing number of studies on SNPs has led to the creation of several specialized databases: (*i*) dbSNP (Sherry et al., 2001), (*ii*) SNPedia (Cariaso and Lennon, 2012), (*iii*) NHGRI-EBI GWAS Catalog (Sollis et al., 2023), (iv) SNPCurator (Tawfik and Spruit 2018), (*v*) miRNASNP-v3 (Liu et al., 2021), and (*vi*) dbGSRV (Li *et al.,* 2022).

Although public SNP databases are regularly updated, there is a lack of information on specifics, such as bibliographic references, the genome region where the SNPs are located, and the registration of polymorphisms described in articles. Therefore, the development of a specialized SNP database for human viruses complements available databases, as it provides details regarding specific conditions of severity, susceptibility, and other aspects.

In response to the growing number of studies investigating the association between human SNPs and viral infections, we developed HVSnp - a manually curated database that integrates information from peer-reviewed literature and public repositories. HVSnp provides structured, high-quality data on human genetic variation linked to viral pathogenesis, aiming to support research on host susceptibility, disease mechanisms, and the development of targeted interventions.

## Material and Methods

### Virus selection and SNP sources

Firstly, we list the most common human viruses, that included: “Adenovirus”, “Chikungunya virus”, “Cytomegalovirus”, “Dengue”, “Ebola virus”, “Epstein-Barr”, “Influenza”, “Hepatitis (A, B, C, D), “Herpes”, “HIV”, “Measles”, “MERS”, “Mumps”, “Parainfluenza”, “Papillomavirus”, “Respiratory syncytial virus”, “Rhinovirus”, “Rubella”, “SARS”, “SARS-CoV-2”, “Yellow fever”, “West Nile virus” and “Zika virus”.

We looked for the virus previously listed in SNPedia (Cariaso and Lennon, 2012), and the SNPs found were the first information in our database construction. The additional information was obtained in dbSNP (Sherry et al., 2001) and Ensembl (Howe et al., 2021). The SNPs were also mined in articles using PubMed and Google Scholar. In addition, the articles served as references to the summarized details for each SNP.

### Inclusion criteria and manual curation

The criteria for registering the SNP were that the SNP researched in association with viruses had to be listed at least in one article (research article, brief or short communication, or review), in methodology (main text or supplementary material), and/or in the results section. The SNPs and articles that did not fit the criteria were excluded. The data mining was done manually and designed to improve the accuracy in HVSnp.

### Database construction and content

The relational database used was divided into tables. Each table corresponds to one viral disease. We based the obligate SNP information on preexisting SNP databases, mainly, dbSNP (Sherry et al., 2001), SNPedia (Cariaso and Lennon, 2012), and SNPCurator (Tawfik and Spruit, 2018). We included three categories: Gene Function, Trait, and Summary. The categories are enumerated in Table 1. 

**Table 1 -:** Mandatory information about the SNPs recorded.

Category	Summary
Id	Reference SNP (rs) identification
Orientation	Plus/ minus
Reference alleles	Ancestral alleles
Alternative alleles	Non-risk alleles
Risk alleles	Alleles that affect character or disease
Gene	Gene or position where the SNP is localized
Gene Name	Complete gene name
Gene Function	Data provided from STRING (Szklarczyk et al., 2021) or GeneCards (Safran et al., 2021)
Chromosome	Chromosome of SNP
Position	Chromosome position
Trait	SNP effect on disease
Summary	Short description of the SNP-related article
Population	Population or ethnicity studied
Publication	References indicated by Pubmed id

We entered the data from Table 1 according to the following steps: first, we identified the SNP with the SNP reference number (rs) in the article; then, we collected information for the summary, population, and article accession, trait, alleles, chromosome position, genes, and their respective functions (obtained by String (Szklarczyk et al., 2021) or GeneCards (Safran et al., 2021)). In cases where details were missing in the article, we searched for SNP specifications in dbSNP (Sherry et al., 2001) or Snpedia (Cariaso and Lennon, 2012).

### Database architecture and implementation

We used the relational database system MySQL (version 8.0) and the Google Sheets architecture in the HVSnp management. The database web page was created in Looker Studio and can be accessed at https://lookerstudio.google.com/s/pGUC7m538gQ.

## Results and Discussion

### Database contents

HVSnp currently comprises curated data on 18 human viruses, encompassing 1,691 unique SNPs mapped across 651 genes, resulting in a total of 2,020 annotated records. The number of SNPs recorded by each virus is: Adenovirus (17 SNPs), Chikungunya (7), COVID-19 (314), Cytomegalovirus (54), Dengue (62), Epstein-Barr (54), Hepatitis A (3), Hepatitis B (380), Hepatitis C (166), Herpes (8), Human immunodeficiency virus (479), Papillomavirus (87), Influenza (74), Measles (68), Respiratory syncytial virus (50), SARS-CoV (80), West Nile (68), and Zika (49) (Figure 1a), totaling 2020 records. 

**Figure 1 -:**
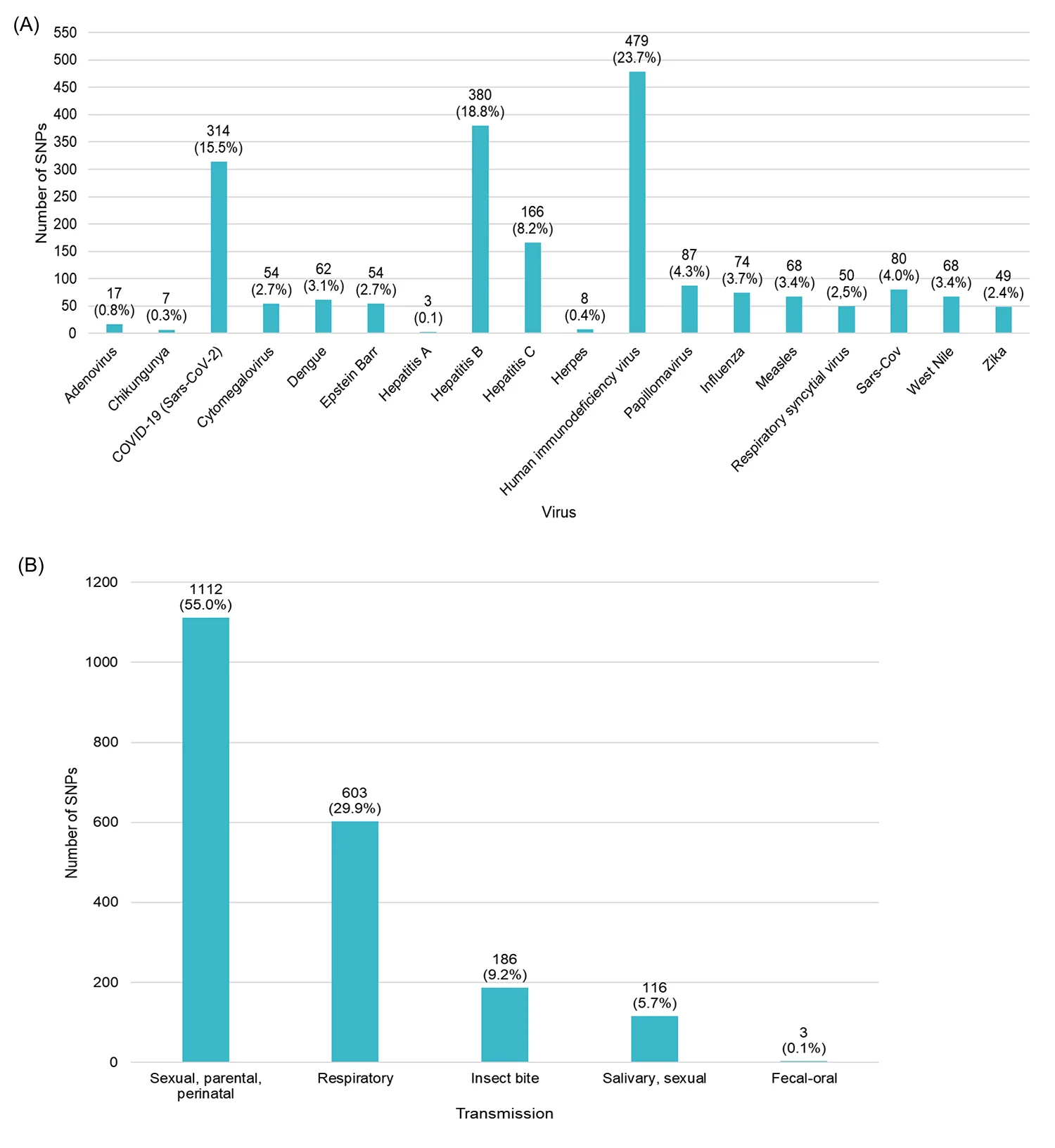
Database metrics. (A) Number of SNPs recorded by each virus. (B) Number of SNPs divided by transmission route.

Several SNPs appear related to more than one virus. For example, rs1800896 was the most frequent SNP studied to: Cytomegalovirus (Kasztelewicz et al., 2017), Epstein Barr (Ghesquières et al., 2013), Hepatitis B (HBV) (Truelove et al., 2008), Hepatitis C (HCV) (Naeemi et al., 2018), HIV (Sezgin et al., 2010), Papillomavirus (HPV) (Torres-Poveda et al., 2012), Respiratory syncytial virus (RSV) (Holster et al., 2018), SARS-CoV (Chong et al., 2006), and ZIKV (Santos et al., 2023). This SNP is located in the gene Interleukin 10 (IL-10), which prevents immunopathology caused by inflammatory responses, and it is fundamental during acute infection and the mechanisms of viral persistence (Rojas et al., 2017).

### Descriptive statistics

Viruses propagate in different ways. We classified the 18 viruses by transmission and summed the total of SNPs for each mode, as observed in Figure 1b. We found 1112 SNPs in association with sexually, parental, and perinatal transmitted viruses, including HBV, HCV, HIV, and HPV. (Table S1). In agreement with the World Health Organization (WHO), HIV, viral hepatitis, and other sexually transmitted diseases cause 2.3 million deaths and about 1.2 million cancer cases annually, and persist as a major public health burden worldwide. Besides, 1 million people are newly infected with sexually transmitted infections daily, and 4.5 million with HIV, HBV, and HCV each year (WHO, 2022). These data explain the relevance of studies associated with sexually transmitted viruses.

Regarding the genes recorded, Syntaxin binding protein 5 (STXBP5) was the most frequent gene recorded, with 83 matches, with all SNPs studied in association with COVID-19. The results indicated that SNPs within *STXBP5*/*STXBP5-AS1* and their interaction may elevate the risk of death in COVID-19 individuals (Hu et al., 2021). 

### User interface and comparative evaluation

The HVSnp Home page with the 18 diseases, with hyperlinks, directs to each disease page. Figure 2 shows the Home page. The search starts when the user selects the disease of interest on the Home page. The database will open the table content of the disease, which has SNPs with their information recorded: ID, Orientation, Reference alleles, Alternate Alleles, Risk Alleles, Gene, Gene Name, Gene function, Chromosome, Position, Summary, Population, and Publication (Figure 3). The user can export the table in CSV or Google Sheets format.

**Figure 2 -:**
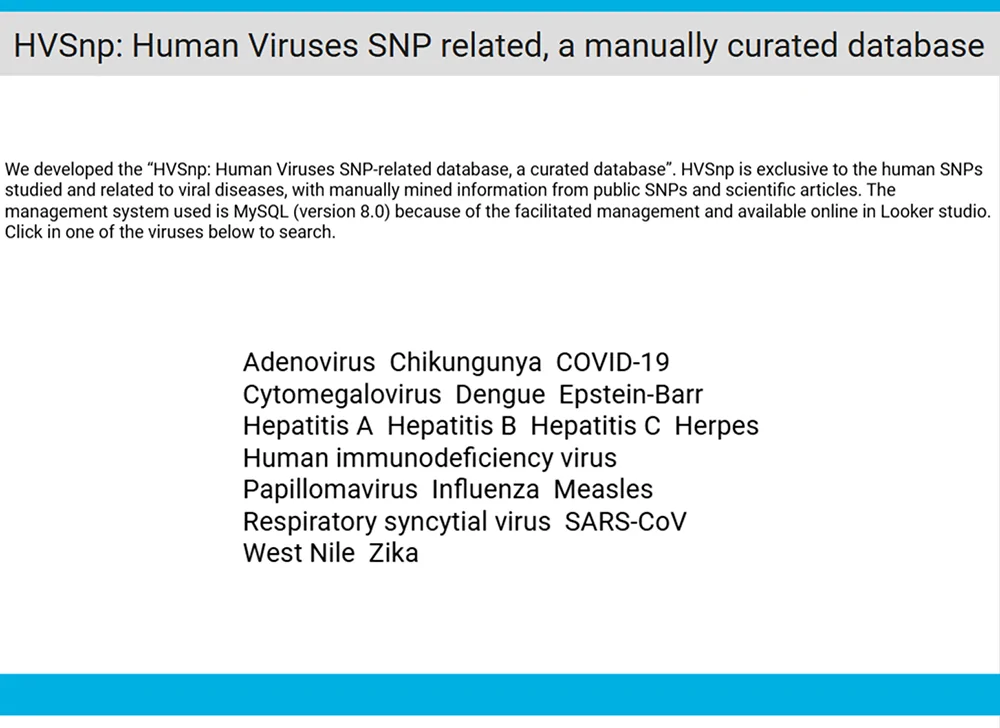
HVSnp interfaces. Home page interface.

**Figure 3 -:**
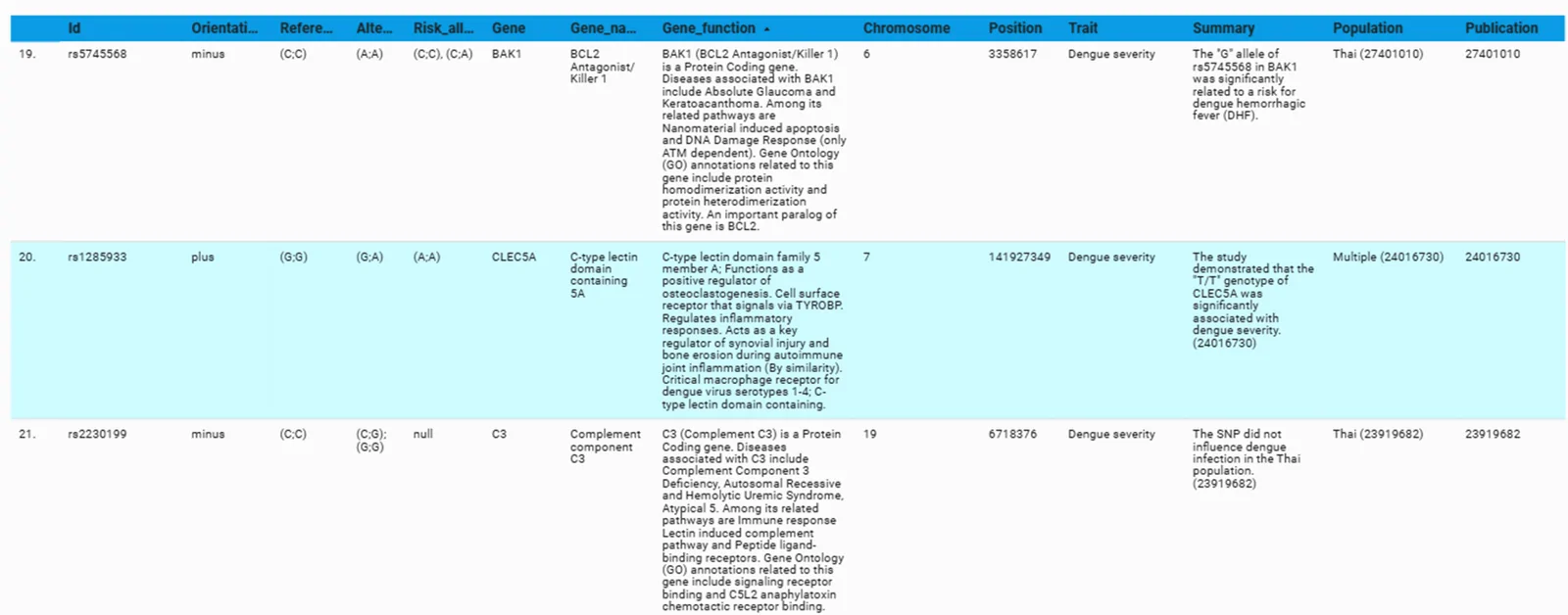
HVSnp interfaces. Representation of the dengue searching with SNPs and respective annotation.

In comparison with dbSNP (Sherry et al., 2001), SNPCurator (Tawfik and Spruit, 2018), and SNPedia (Cariaso and Lennon 2012), HVSnp has a lower number of SNPs, as these databases contain more traits and diseases related to polymorphisms. HVSnp is similar to SNPCurator (Tawfik and Spruit, 2018), but it uses an automated text-mining tool to search for information only in the abstracts of the papers, whereas HVSnp is manually curated, and the full text is analysed to search for a SNP association. dbGSRV (Li et al., 2022) is a manually curated database that includes SNPs related only to respiratory viruses, unlike HVSnp, which encompasses not only respiratory viruses but also sexually transmitted, parental, perinatal, insect bite, and fecal-oral transmitted viruses. In addition, the Summary could help users, once it explains the consequences of one SNP to the viral disease, following the article’s results.

HVSnp has some limitations. The analyses that relate human SNPs and viral diseases are less common, when compared with cancers and metabolic disorders, for example, some viruses did not show studies relating them to human viruses; and the majority of the studies used WES or candidate genes, not GWAS, which indicated the restricted number of samples analyzed. We intended to update the HVSnp annually to improve the data.

## Conclusion

HVSnp is a database with human SNPs studied for 18 viral diseases. In total, 1691 unique SNPs were recorded in HVSnp, and it was observed that one SNP can be associated with more than one viral disease, including susceptibility and severity, for example. The SNP data provides the essential information between SNPs and disease on a user-friendly site. In the future, we aim to expand the database with more diseases and SNPs to update the HVSnp.

### Supplementary material

The following online material is available for this article:

Table S1 - Virus with the number of SNPs related and classified by transmission.

## Data Availability

The HVSnp is available online: https://lookerstudio.google.com/s/pGUC7m538gQ.
