# Supplementary material for: HVSnp: A manually curated database of human SNPS associated with viral infections
Source: Genet Mol Biol. 2026 Oct 19;49(3):e20250075. doi: 10.1590/1678-4685-GMB-2025-0075 (PMC13679873; doi:10.1590/1678-4685-GMB-2025-0075)
Supplement: Supplementary file 1 [file 1415-4757-GMB-49-3-e20250075-s1.pdf]

Genetics and Molecular Biology
https://doi.org/10.1590/1678-4685-GMB-2025-0075

Supplementary Material to "HVSnp: A manually curated database of human SNPS associated with viral infections"

Table S1 - Virus with the number of SNPs related and classified by transmission.

**Diseases**	**Transmission**	**Total of SNPs**
Adenovirus	Respiratory	17
Chikungunya	Insect bite	7
COVID-19	Respiratory	314
Cytomegalovirus	Salivary, sexual	54
Dengue	Insect bite	62
Epstein Barr	Salivary, sexual	54
Hepatitis A	Fecal-oral	3
Hepatitis B	Sexual, parental, perinatal	380
Hepatitis C	Sexual, parental, perinatal	166
Herpes	Salivary, sexual	8
Human immunodeficiency virus	Sexual, parental, perinatal	479
Papillomavirus	Sexual, parental, perinatal	87
Influenza	Respiratory	74
Measles	Respiratory	68
Respiratory syncytial virus	Respiratory	50
Sars-Cov	Respiratory	80
West Nile	Insect bite	68
Zika	Insect bite	49
Total		2020
